# Carer Empowerment Is Key to Reduce Dementia Care Inequalities in the Middle East

**DOI:** 10.3390/ijerph18084378

**Published:** 2021-04-20

**Authors:** Syed Fahad Javaid, Aishah Al-Zahmi, Munir Abbas

**Affiliations:** 1 Department of Psychiatry and Behavioral Sciences, College of Medicine and Health Sciences, United Arab Emirates University, Al Ain P.O. Box 17666, United Arab Emirates; 2 Behavioral Sciences Institute, Al-Ain Hospital, Al-Ain P.O. Box 1006, United Arab Emirates; azahmi@seha.ae (A.A.-Z.); Munirabbas222@gmail.com (M.A.)

**Keywords:** dementia, Middle East, carers, health inequality

## Abstract

Dementia represents a significant problem in the Middle East. Sociocultural and political factors that shape conceptions of health and care tend to stifle research and the dissemination of knowledge throughout the Middle East. These socio-political challenges concerning engagement with individuals living with dementia and their carers include language barriers, stigmatization, logistical constraints, lack of informal support outside of hospitals, and over-dependence on clinicians for dementia information. There is an urgent need in the Middle East to increase care and support for adults with dementia and their carers, enhance research efforts and improve the dissemination of information related to dementia in the region. One possible way to do so is to begin to promote a knowledge-based culture throughout the Middle East. This can be achieved by aligning traditional deterministic and spiritual perspectives of mental health with more Western, scientific, and evidence-based models. We suggest employing practical, multidimensional approaches to deal with the stated challenges, both at individual and societal levels. Doing so will improve knowledge of dementia and allow health and social care systems in the Middle East to begin to address a growing problem.

## 1. Background

Dementia represents a significant problem in the Middle East [1]. According to recent estimates, approximately 1.2 to 2.3 percent of individuals aged 50 and older and 13.5 to 18.5 percent of adults aged 80 and over have diagnosable dementia [2]. These figures may be underestimated due to underreporting of dementia cases and low access to health and social care services by a large proportion of the population [1]. Both micro- and macro-level factors exist within Middle Eastern culture that serve as barriers to conducting research and contributing new knowledge to the understanding of dementia [3]. Sociocultural and political factors that shape conceptions of health and care tend to stifle research and the dissemination of knowledge throughout the Middle East, which can have negative impacts on the ability to understand the health needs of the population [3].

These socio-political barriers can lead to challenges with regard to engaging with individuals living with dementia and their carers [3]. Much of the population resides in rural locations with limited access to telecommunications [4]. Physical contact is needed to reach carers in these settings, and this can present a logistical challenge when conducting research related to dementia [3,4]. Environmental factors, such as extreme heat and undeveloped roads, can reduce the ability to contact carers in many parts of the region [4]. There appears to be an awareness deficit associated with dementia in Middle Eastern countries, and a need does exist to consistently and accurately evaluate trends in this mental health issue over time [1].

There is no apparent financial basis for inherent inequities with regard to the quality of care available to elderly citizens in the Middle East. Healthcare is free for citizens of most countries in the region. However, there are known differences in the utilization of clinical services by the large expatriate population compared to the citizens. According to one study in Abu Dhabi, UAE citizens on average used outpatient clinical services once per month compared to expatriates, where usage rates were 3–4 times less [5]. Inconsistent insurance cover for mental health services utilized by the expatriate population worsens the situation [6]. Inequities also exist in public health and knowledge dissemination about dementia, as there is currently a knowledge scarcity pertaining to this mental health condition throughout much of the Middle East [1]. However, further research is still needed regarding the extent to which these inequities influence dementia incidence and the quality of care provided.

## 2. Barriers to Accessing Dementia Care

A major problem faced by individuals living with dementia and their carers is stigmatization [7]. A stigma, or the process of stigmatization, refers to a sense of shaming or externally induced embarrassment regarding some characteristic [8]. Broad social and cultural shaming of mental health issues can reduce help-seeking behaviors and may cause embarrassment to adults with dementia, their families, and carers [4]. The prevailing culture in the Middle East is collective in nature, with an emphasis on community and family opinions compared to individual viewpoints. Public image is seen as extremely important. Consequently, the social stigma of having a mental illness is a major obstacle for patients and carers seeking appropriate support and treatment [9]. These stigmas have been potentially magnified in the midst of the COVID-19 pandemic. Evidence suggests that COVID-19 has exacerbated many of the problems and barriers that exist for carers in the Middle East, including with respect to avoiding seeking out health care services [10,11]. In the Middle East, where much of the population resides outside of large urban centers with limited access to mental health care services, the constraints associated with COVID-19 have further impeded the ability of many carers to receive the support that they need [10,11,12].

The Middle Eastern culture has a differing perception of health and the causes of mental and physical illness than in the West [7]. In the Middle East, a more spiritual and deterministic view of health and illness is used [13,14]. This attitude potentially contributes to stigmatization and reduces help-seeking behaviors [8]. Additional obstacles in studying dementia perceptions and engaging with dementia carers in the Middle East include language barriers, a lack of knowledge, lack of informal support outside of hospitals, and over-dependence on clinicians for dementia information [1]. These factors contribute to the centralized control of information and prevent evidence from quickly reaching the population to respond to dementia care needs [1]. There are approximately 60 different languages spoken in the Middle East. Reaching out to living with dementia and their carers who speak different languages produces communication challenges that can result in a limited ability to provide support [14].

## 3. Challenges in Dealing with the Problem

The diversity of the Middle East makes a unified and coordinated strategy for addressing these barriers particularly difficult [14]. This article pertains to the Middle East in general, and no primary data were collected regarding the socio-cultural context with which dementia exists. However, secondary data from prior peer-reviewed studies have been used to support the arguments being made [15]. Religion is a major common factor that binds various Middle Eastern sub-cultures. In a traditional Muslim family, religion acts as the first reference to understand illnesses, such as dementia, and is used as a guide to deal with the issues [15]. A critical step, therefore, in the advancement of knowledge and care related to dementia in the future is to align traditional deterministic and spiritual and religious perspectives of mental health with more Western, scientific and evidence-based ones so that treatments can be applied to older Middle Eastern population members in accordance with their views on health care [16,17]. An abundance of recent research has already begun to demonstrate ways in which this can be done effectively [14,15,16,17]. Arabic language is rich with metaphors. This is evident in the verses of the Islamic holy text which includes many metaphoric directives [16]. Research supports the use of more figurative language to support person-centered care in the case of dementia, as well as takes into account Islamic perspectives of the role of the environment in the provision of nursing care [16,17].

There is an urgent need in the Middle East to increase care and support for individuals living with dementia and their carers. One possible way to do so is to begin to promote a knowledge-based culture throughout the Middle East. Through encouraging education and information dissemination, many of the barriers to dementia knowledge can be uprooted. Translation of research-based evidence into different languages spoken in the Middle East and encouraging health and social care practitioners to develop some proficiency in multiple languages may help overcome some of the access issues in communication. Socio-political strategies to encourage the sharing of Western and Eastern research related to dementia and decentralize the control of research and information within health and social care are also necessary to begin to promote access to persons living with and their carers. Finally, increased infrastructure to establish telecommunication networks in rural regions in the Middle East may improve access to carers and patients living outside of large, urban settings.

## 4. Suggested Solutions

Our experience, both at local clinical and research fronts, has confirmed the presence of the stated obstacles to accessing dementia carers’ views. There are inequalities in access to and use of formal memory and mental health care, such as using support groups, receiving help from community mental health teams, and accessing hospital and inpatient support. However, collaborative working with local community leaders, health and social care authorities can help to effectively support the carers.

The region’s large expatriate community needs to be included in any efforts to improve the experience of carers looking after persons with dementia. This population group will benefit from services such as access to dedicated dementia support groups, expanded insurance cover, and a helpline in different languages. This will, in turn, improve researchers’ access to this important yet underserved section of the population.

We suggest the following practical strategies to empower dementia carers in the Middle East: (a) Working in cohesion with well-established local community mental health teams. (b) Developing unambiguous dementia information literature in Arabic with an option to contact an Arabic-speaking professional to have further discussion. To improve carers’ access to information, we suggest forming a body of experts from health, social care, and legal domains to work with individuals living with dementia and their carers to agree on accurate, culturally relevant information that carers can use. Carers will benefit from information on their rights, available services, and the importance of maintaining their health and social standing. (c) Organizing knowledge-based events for local primary care clinicians about supporting dementia carers’ access to appropriate care. (d) Maximizing carer convenience by offering telephone and web-based consultations at the time of their preference. (e) Using traditional and social media platforms to approach dementia carers with opportunities to volunteer and contribute to carer support groups.

## 5. Conclusions

In conclusion, persons living with dementia and their carers in the Middle East face inequalities in accessing support and care. The causes are diverse and varied, including stigma, language barriers, lack of informal care outside of the hospital environment, and an over-dependence on clinicians for information. Each of these factors also reduces the opportunity to conduct research related to dementia in the Middle East. Hence, there is a lack of research on the perspective of individuals living with dementia and their carers in the region, and more work is needed to explore the views of this population. A possible solution lies in a multidimensional approach that involves micro- (e.g., health systems, telecommunications) and macro- (e.g., sociocultural change) strategies. Doing so will improve knowledge of dementia and allow health and social care systems in the Middle East to begin to address a growing problem.

## Data Availability

Not applicable.
